# A Contemporary Review of Evidence for Transoral Robotic Surgery in Laryngeal Cancer

**DOI:** 10.3389/fonc.2018.00121

**Published:** 2018-04-18

**Authors:** Philippe Gorphe

**Affiliations:** Department of Head and Neck Oncology, Institute Gustave Roussy, Université Paris-Saclay, Villejuif, France

**Keywords:** transoral robotic surgery, laryngeal neoplasms, TORS, supraglottic laryngectomy, total laryngectomy, glottic cordectomy

## Abstract

Numerous studies have shown that transoral robotic surgery (TORS) for oropharyngeal cancers is safe and that it yields satisfactory functional and oncological outcomes. For many teams worldwide, it is therefore a standard surgical approach with eligible patients. In the same time, TORS is increasingly being used and described in the context of laryngeal cancer surgery. It is proposed as an alternative to open approaches, which may yield inconsistent functional results and significant rates of postoperative complications. It may also be an alternative to definitive radiotherapy, which entails significant early and late toxicities. Moreover, it has been explored as an alternative to endoscopic laser surgery in patients with difficult exposure, even though there is still a lack of evidence about which procedure provides better vizualization of the vocal cords. This article provides a review of the indications for TORS in laryngeal cancer, the peri-operative morbidity, functional outcomes, and oncological results.

## Introduction

The underlying principle of transoral robotic surgery (TORS) is to be able to reliably perform state-of-the-art oncological resection of the primary tumor through a minimally invasive transoral approach (1). Surgery is assisted by remote-controlled miniaturized surgical instruments and magnified visualization with a high-definition three-dimensional camera. It has proven to be an effective alternative to open surgery, with or without a mandibulotomy approach for oropharyngeal cancer in a large number of studies since its approval by the FDA in 2009 for T1 and T2 lesions. Series showed decreased rates of postoperative complications, improved functional outcomes, and favorable oncological results (2). For a large number of authors, it has become a standard-of-care among other treatment modalities in oropharyngeal squamous cell carcinoma staged T1–T2 (1, 3–5). With laryngeal cancers, TORS has been explored as minimally invasive surgery for supraglottic and glottic lesions, as well as for total laryngectomy. This article provides a review of the evidence available to date in regard to indications for TORS in laryngeal cancer, peri-operative morbidity, functional outcomes, and oncological results.

## Transoral Robotic Supraglottic Laryngectomy

### Indications

Supraglottic laryngectomy accounts for the vast majority of transoral robotic surgical procedures published in regard to laryngeal cancer. Transoral robotic supraglottic laryngectomy (TORS-SGL) must allow for complete oncological resection of the supraglottic tumor, while it must also preserve anatomical and neurophysiological functions of the glottic larynx (i.e., protective, respiratory, and phonatory functions) and of the base of the tongue. For these reasons, the preferential indications are selected T-stage T1 and T2, and a few T3 cancers (6). The three following groups of tumors have been reported to be potentially suitable for open supraglottic laryngectomy; TORS-SGL must be assessed for each group according to their specific local extensions (7).

The first group encompasses the early T-stage tumors of the anterior epilarynx (suprahyoid epiglottis) and lateral epilarynx (laryngeal aspects of the aryepiglottic folds). The extent of resection in transoral procedures depends mainly on local invasion of the vallecula and of the lateral pharynx through the threefold region. Small tumors originating from the antero-lateral epilarynx are amenable to partial surgery with good functional outcomes and local control. When the hyoid bone is not directly involved with tumor and the vallecula mucosa and tongue base are uninvolved, the feasibility of hyoid bone preservation has been demonstrated in literature from open supraglottic laryngectomy, from transoral laser microsurgery (TLM), and from TORS-SGL (6, 8–10). Tumors of the posterior epilarynx (arytenoids) carry an increased risk of postoperative aspiration and swallowing difficulties. They are challenging cases and they should therefore only be performed by the most experienced surgeons. The second group includes tumors of the anterior laryngeal vestibule (infrahyoid epiglottis). In these tumors, the risk of local extension into the pre-epiglottic space (PES) argues for complete resection of this space (11). Moreover, tumors of the infrahyoid epiglottis may extend superficially to ventricular bands and they may require their resection along with resection of the paraglottic space above the plane of the ventricle. In the third group that comprises the early T-stage tumors of the vallecula (12), surgeons need be careful when considering TORS-SGL. Potential local extensions that are challenging for a transoral robotic approach are deep involvement of the muscles of the base of tongue, and lateral submucosal extensions to the pharyngoepiglottic and aryepiglottic folds. Frozen sections should be routinely performed to ensure complete resection.

In the literature to date, 35% of the published patients operated on by TORS-SGL with available clinical staging were reported to have a cT1 disease, 53.5% had a cT2 disease, and 11% had a cT3 cancer (13–25). Twenty-three patients were reported to have been operated on for salvage surgery after radiotherapy (22, 25, 26). Few details were provided in series regarding the precise disease location and the extensions for each patient, although the epiglottis was involved in the vast majority of the patients with an available clinical description. Unfortunately, for most of the patients it was not possible to distinguish between suprahyoid tumors, infrahyoid tumors, and tumors that involved both sites of the epiglottis.

### Contraindications

Absolute contraindications to TORS-SGL comprise: insufficient transoral exposure (e.g., interincisor distance <3cm, trismus, and difficult exposure); invasion of the thyroid and/or cricoid cartilage; impaired vocal cord mobility or arytenoid mobility; invasion of the paraglottic space; posterior commissure invasion; extension to the glottic larynx; involvement of more than 2 cm of the base of the tongue mucosa or invasion of the tongue base muscles. Relative contraindications to TORS-GL include pulmonary disease and a respiratory insufficiency given the risk of postoperative aspirations. Moreover, elderly patients should be carefully evaluated for their performance status.

### Surgical Procedure

The following three steps are the main components of the standard complete TORS-SGL for a supraglottic laryngeal cancer (13, 27). They can be performed in any order, provided that exposure remains adequate and allows for a complete visualization of the tumor for oncological monobloc resection (25). For tumors of the vallecula, a transvallecular approach is to be replaced by incision of the tongue base. Weinstein et al. recommend beginning with splitting of the suprahyoid epiglottis, sectioning the epiglottis vertically up to the petiole, then performing each side of the supraglottic laryngectomy one by one (13).

A transvallecular approach to the PES is initiated by transection of the vallecular mucosa, identification of the hyoid bone, dissection of the thyro-hyoid membrane, and identification of the thyroid cartilage. Laterally, superior laryngeal vascular bundles are encountered in the dissection of the lateral pharyngoepiglottic folds, and they are clipped. Dissection of the PES is then performed.

Aryepiglottic folds are transected superiorly and laterally to the arytenoid. The dissection is pursued between the arytenoid cartilages and ventricular bands to the level of the ventricles, allowing resection of the false vocal cords and the upper paraglottic spaces above the ventricles within the specimen.

Horizontal sectioning goes through the ventricles, exposing the ventricular floors, and the vocal cords. Anteriorly, the petiole is transected just above the anterior commissure, and the transection is connected to the dissection of the lower PES.

**Figure 1:**
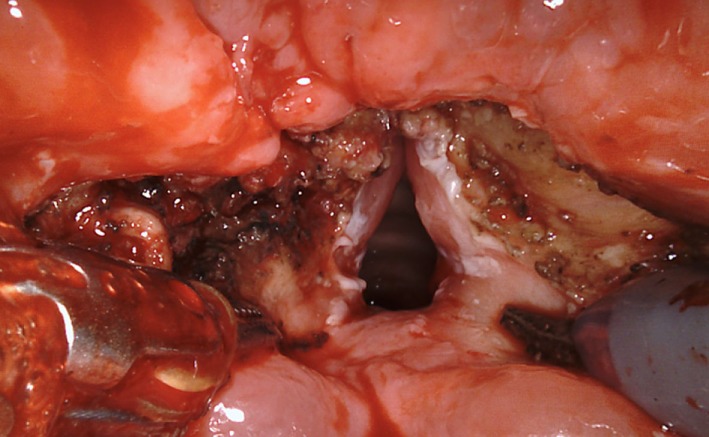
Transoral robotic supraglottic partial laryngectomy in a patient with epiglottic cancer.

After completion of these three steps, the specimen can readily be removed and the margins are determined from frozen sections if necessary (Figure 1). In almost all of the cases reported in literature, the patient selection and the exposure were sufficient to ensure complete oncological resection *via* the transoral robotic approach. Only two patients were reported to have been converted to an open approach due to limited exposure and dissection (19, 25). Neck dissections can be performed during the same procedure. For some authors, neck dissections are performed secondary within the first 3 weeks following the supraglottic laryngectomy, to limit the edema and the need for a tracheotomy (13, 18, 21, 28–30).

### Peri-Operative Outcomes

The reported rates of postoperative complications are low. Mendelsohn et al. reported 5 instances of temporary vocal cord hypomobility out of 18 patients (17); Lallemant et al. reported bleeding at day 2 in 1 patient out of 23 patients (16); Alon et al. reported a skin thermal injury with late laryngeal stenosis (14); while Kayhan et al. reported two postoperative pulmonary infections and one late laryngeal stenosis (21).

In several series, extubation of patients occurred at least 25 h after the surgery (18, 21, 22). TORS supraglottic laryngectomy could be performed without a tracheotomy in most of the patients. In the largest series published to date, only 12 patients out of 84 (14%) required a per-operative tracheotomy (25). However, eight patients required a secondary tracheotomy for postoperative edema. Only 1 tracheotomy out of 84 patients was definitive. The management of postoperative nutrition varies between authors; some authors have published oral feeding as soon as day 1 (19); however, the postoperative period was generally assisted with nasogastric feeding, and for most of authors oral intake was resumed between 7 and 10 days on average (13, 16, 17, 25, 31).

### Oncological Outcomes

When reported, the rate of complete resection with free margins was between 60 and 100% in series comprising at least 10 patients (16, 17, 20, 25, 31, 32). The rates of postoperative radiotherapy ranged from 40 to 70% (16, 17, 20, 25, 31, 33). The rates of local recurrence when reported ranged from 0 to 11% (34). The 2-year overall survival were reported in two series and ranged from 66.7 to 88.9% (15, 17).

## Transoral Robotic Total Laryngectomy

### Indications

Transoral robotic total laryngectomy (TORS-TL) has been shown to be feasible, although only a very small number of patients have been operated on by this method to date (35–38). While yet to be proven, the reported objective is to decrease postoperative morbidity; first, by decreasing the risk of postoperative fistula as a result of better preservation of the pharyngeal mucosa; secondly, by limiting the lateral dissection between the pharyngeal and vascular spaces, thus decreasing the risk of a carotid blow-out. However, the vast majority of patients who require a primary total laryngectomy present with a locally advanced squamous cell carcinoma of the larynx, and they therefore require a bilateral neck dissection concurrent with the laryngectomy. The benefits of a minimally invasive approach to the laryngeal site in these patients are therefore somewhat questionable, and precise indications have yet to be well-defined.

The small number of surgeons experienced with this procedure recommends that TORS-TL be evaluated in the following three indications where neck dissections can be avoided (35–38). The first indication is salvage surgery for locally limited failure of the primary lesion after radiotherapy or chemoradiotherapy that is not amenable to a salvage partial laryngectomy due to local or general contraindications. Naturally, the primary pharyngeal closure must be readily achievable and there should not be a need for a flap closure. Preservation of the infrahyoid muscles must be oncologically possible without incurring risk, meaning that there must not be any doubt about integrity of the thyroid cartilage or the cricothyroid ligament. In practice, this situation is rare in a salvage context. The second and even rarer indication is for benign or malignant laryngeal tumors with limited local extensions requiring a primary total laryngectomy for oncological or functional reasons, while not requiring extensive perilaryngeal dissection. Potential rare and various histologies encompass adenoid cystic carcinoma, low-grade chondrosarcoma, and chondroma, among others. The third indication is refractory laryngeal dysfunction with long-term tracheotomy and enteral feeding. Such indications are encountered in patients with a neurodegenerative disease, in patients with definitive high-grade sequelae from a previous laryngeal trauma who generally have undergone multiple operations prior to the laryngectomy decision, and in patients with severe chronic post-radiotherapy toxicity, with or without chondronecrosis. However, careful patient selection is required in light of the potential for associated pharyngeal dysfunction, as a pharyngeal stenosis would not be improved without mucosa repair with a flap.

Transoral robotic total laryngectomy has been reported in the literature for series that comprised multiple cancer localizations and various robotic surgical procedures (39). However, only three publications to date have comprised series of patients operated on with TORS-TL, with the total number of patients limited to 15 (36–38). Notably, two patients eventually had unsuccessful exposure for complete robotic resection. Six patients (40%) had a local squamous cell carcinoma recurrence after radiotherapy; three patients (20%) had a primary surgery for a rare endolaryngeal tumor, comprising two low-grade chondrosarcoma and one cystic adenoid carcinoma; and six patients (40%) had a functional total laryngectomy for a chronic laryngeal dysfunction, due to sequelae from radiotherapy in three patients, a neurodegenerative disease in one patient, an idiopathic bilateral vocal cord palsy in one patient, and due a multioperated laryngeal stenosis in another patient.

### Procedure

The first part of the procedure is performed *via* a minimal open approach of the neck. A skin excision is made in front of the future stoma, and the thyroid isthmus is divided. The trachea is sectioned, the tracheal part of the specimen is dissected from the esophagus to facilitate its superior mobilization during the transoral dissection, and the inferior side of the stoma is sutured.

**Figure 2:**
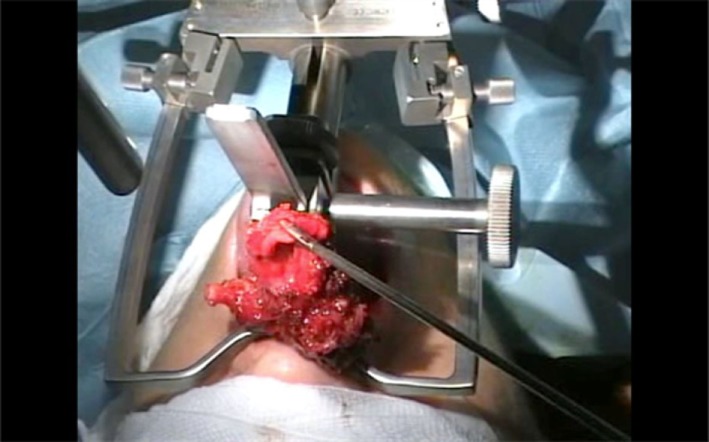
Transoral removal of the larynx with the robot moved out of position and the Faye–Kastenbauer mouth retractor in place.

The second part of the procedure begins as a transoral robotic supraglottic laryngectomy. The pharyngoepiglottic folds are divided, superior laryngeal vascular bundles are clipped, and the mucosa of the vallecula is incised. This allows for exposure of the superior side of the hyoid bone, which is resected within the specimen in TORS-TL. The anterior side of the hyoid bone is then followed, and dissection of the pre-epiglottic fat may or may not be carried out according to the surgical indications. The thyro-hyoid membrane is dissected, until the upper side of the thyroid cartilage can be identified. The thyroid alas are freed by dissection and moved posteriorly, while preserving the outer perichondrium along with the infrahyoid muscles. On each side the anterior pyriform sinus mucosa is incised as close to the larynx as possible and the lateral pyriform sinus is mobilized, allowing dissection of the constrictors after transection of the lateral thyro-hyoid ligaments (laryngeal suspensory ligaments). Finally, the postcricoid mucosa is sectioned under direct transoral visualization, and the specimen is freed *via* the oral cavity (Figure 2). The pharynx is closed with a single horizontal suture line if possible, although it can also be sutured to the preserved strap muscles in a U-shaped configuration if necessary. Of note, Krishnan et al. have reported that they prefer removing the larynx through the cervical opening rather than through the oral cavity (38). In their opinion, transoral delivery of the larynx was associated with dilatation of the pharyngeal defect and it resulted in a larger neopharynx to be closed.

A technical limitation of TORS-TL that has been reported by several authors is exposure of the larynx during the transoral procedure. Exposure has been described in some cases to be insufficient. In the series of Dowthwaith et al., one patient out of three had an inadequate transoral exposure (36); in the series of Smith et al., two patients out of seven had an inadequate transoral exposure (37); while Krishnan et al. did not report any difficulty among five patients (38).

### Peri-Operative Outcomes

Postoperative pharyngocutaneous fistulas were reported in three patients: two patients underwent salvage TORS-TL after failure of radiotherapy, and one patient underwent a functional TORS-TL for a multioperated laryngeal stenosis (37, 38). The rate of postoperative pharyngocutaneous fistulas was 0–28.6% (36–38). One patient in the series of Dowthwaith et al. had bleeding of the pharyngeal suture on day 9 after resumption of oral intake, and he required transoral cauterization (36). Other patients in their series resumed oral intake on day 7 and day 8. In the series of Krishnan et al., oral intake was resumed on day 10 and day 12 for the two patients who were not percutaneous enteric gastrostomy dependent for nutrition prior to the TORS-TL (38).

### Oncologic Outcomes

Krishnan et al. reported free margins for the three patients operated on due to a cancer in their series. The first patient had an rT2N0M0 squamous cell carcinoma, the second patient had an adenoid cystic carcinoma, and the third patient had a low-grade chondrosarcoma. They were still alive and disease-free 54, 54, and 18 months, respectively, after the surgery.

## Transoral Robotic Glottic Cordectomy

### Indications

Transoral laser microsurgery is established as a standard-of-care for minimal-invasive surgery in early glottic cancer. In supraglottic cancer, it is an alternative that yields similar oncological outcomes to open partial laryngectomy and definitive radiotherapy in selected indications (40, 41). However, adequate exposure of the glottic larynx is sometimes a serious limitation in TLM in a small number of patients with early-stage neoplasms, despite numerous refinements to the procedure by experienced teams (42). The feasibility of TORS in glottic surgery was first demonstrated by O’Malley et al. in 2006 in a canine model (43). In 2009, Park et al. further published the feasibility results for three patients operated on with TORS for a glottic lesion (44); one had a stage rT2N2b glottic carcinoma involving the anterior commissure, one had a stage rT1bN0 recurrent glottic carcinoma after radiotherapy involving the anterior commissure, and one had a glottic melanoma. An improvement of the surgical procedure using CO_2_ fiber was subsequently described by Blanco et al. (45), allowing adequate oncological resection of a T1a glottic carcinoma with good functional outcomes. In 2012, Vural et al. reported a successful TORS glottic resection that removed the complete paraglottic spaces up to the upper border of cricoid cartilage (46). The patient had a recurrent carcinoma of the glottis involving the anterior commissure with impaired vocal cord mobility and a small subglottic anterior extension, 5 years after TLM and adjuvant radiotherapy. The authors referred to the procedure as a supracricoid laryngectomy with preservation of the cartilage framework, because of resection of the paraglottic spaces. However, the epiglottis above the anterior commissure resection was preserved, as was hence the PES. This was the largest glottic procedure case published to date. Since then, four series of patients with early-stage glottic cancer operated on with TORS have been published (16, 32, 47, 48).

In 2012, Kayhan et al. published a series of 10 patients operated on with TORS for T1 glottic cancer (47). The lesions were not described, although they thought to have been stage T1a, as the reported procedure was robotic cordectomy, in patients that were provided therapeutic alternatives including radiotherapy and TLM. Lallemant et al. further described a series of 13 patients (16) operated on with TORS for glottic carcinoma, stage T1a in 6 patients, T1b in 6 patients, and a T2 glotto-supraglottic lesion due to extension to the petiole in 1 patient. The same year, De Virgilio et al. published a review of strategies used at their center to improve TORS exposure (32), including 18 patients operated for glottic carcinoma. Ten patients were stage T1, of whom one had a recurrent disease, and eight patients were stage T2, without providing more details. Recently, Wang et al. described a series of eight patients operated on with TORS for glottic carcinoma with anterior commissure involvement (48), that was either stage T1 (*n* = 3) or stage T2 (*n* = 5, of whom two had a recurrent disease after previous TLM). Therefore, an extension to the anterior commissure appears to be surgically resectable *via* a TORS approach, although the management of thyroid cartilage remains an issue in these patients irrespective of the procedure.

### Peri-Operative Outcomes

All of the patients in the published series had a successful procedure, including adequate exposure and satisfactory resection (16, 32, 47, 48). The frozen sections were free of cancer (47, 48). Kayhan et al. reported that 1 patient out of 10 was tracheotomized for 3 days becdue to chronic obstructive pulmonary disease (47). Lallemant et al. reported one secondary tracheotomy until day 15 after the surgery (16), due to a cervical emphysema and a pneumothorax, along with limited laryngeal bleeding due to a small unnoticed breach of the cricothyroid ligament during anterior commissure resection.

Except for the tracheotomized patient who required a nasogastric tube for 5 days, oral intake was successfully resumed 6–24 h following the surgery in all of the patients for Kayhan et al. (47). In the series of Lallemant et al. (16), aside from the tracheotomized patient who required a nasogastric tube for 18 days, two patients required a nasogastric tube for either 3 or 10 days, and 10 patients resumed oral intake on the first day following the surgery. In the series of Wang et al. (48), including patients with a larger resection that encompassed the anterior commissure, one patient resumed oral intake on the first day following the surgery, while all of the other patients required a nasogastric tube, for a mean duration of 14 days.

### Oncologic Outcomes

Kayhan et al. reported neither the definitive status of the margins nor the postoperative adjuvant treatment; no recurrences occurred during follow-up with a mean duration of 9 months (47). In the series of Lallemant et al., 6 patients out of 13 had a sufficient resection (16), 3 patients had a microscopically positive margin or a close margin of less than 1 mm, and 4 patients had unclassifiable margins due to thermal injury. None of the patients received adjuvant treatment. Two patients out of the 13 (15.4%) had a local recurrence. One local recurrence occurred 10 months after the TORS for a T1b lesion with microscopic margins of less than 1 mm. It was staged rT1 and it was treated with radiotherapy. The other local recurrence occurred 12 months after the TORS for a T1a lesion with unclassifiable margins. It was staged rT4a, and it was treated by a salvage total laryngectomy. De Virgilio et al. reported that all of the margins were free of disease (32). Wang et al. (48) reported that three patients out of eight had microscopically positive margins on the specimen. However, all of these patients were spared postoperative radiotherapy as the definitive pathological examinations of all of the frozen specimens were negative and follow-up examinations did not reveal any suspicious lesions. The mean follow-up period was 40 months, without local recurrence. The 3-year local control, laryngeal preservation, and overall survival were 100%.

## Laryngeal Exposure with the Flex Robotic System

All series reported in the previous sections have been performed using the da Vinci Surgical System (Intuitive Surgical, Sunnyvale, CA, USA). The endowrist instruments and the 30° endoscopic three-dimensional camera are technological improvements that demonstrated very useful for most of transoral procedures. However, the da Vinci System was designed for work in a large cavity as in thoracosopic or laparoscopic surgery, and limitations have been reported when transposed in transoral laryngeal surgery. For Remacle et al., the main difficulty is getting a good vizualization and exposure of the larynx due to the bend around the tongue base, because of rigid robotic arms and endoscopes (49). Furthermore, the limited number of cutting devices is an issue for glottic surgery, notably the lack of available CO_2_ laser fiber (16, 47, 50). The Flex Robotic System (Medrobotics, Raynham, MA, USA) is a new flexible robotic scope that achieved CE mark in 2014 and FDA clearance in 2015. The system consists of a Flex Scope, with flexible instruments inserted through lateral guide tubes along the flexible endoscope and handed directly by the surgeon, and a Flex Console, which controls the position and mobility of the flexible endoscope HD camera and holds the console display (51). The first patients were treated in Europe in June 2014 and the Flex System has demonstrated its feasibility for transoral surgery (51–53). More recently, an European prospective non-randomized multicentric study has established the safety and efficacy of the Flex System for transoral robotic head and neck surgery in 79 patients with a combination of various procedures and lesions (54, 55). Of these patients, 21 underwent TORS with the Flex System for a laryngeal lesion: 11 lesions of the epiglottis, 2 lesions of the ventricular folds, 3 lesions of the arytenoid cartilages, and 5 lesions of the vocal cords. No detail was given regarding the benign or malignant nature of the lesions, nor regarding the procedures performed. Lang et al. reported that the endolarynx exposure was feasible, but that some limitations remained with respect to adequate triangulation for precise resection of smaller lesions inside the larynx (55). Matheis et al. published a focused description of their single-center experience of 40 patients who underwent TORS with the Flex System (54). Sixteen patients had a laryngeal supraglottic lesion, 12 benign and 4 malignant. An oncologic resection was planned and successfully performed in three patients with an epiglottic carcinoma staged T1 (*n* = 1) or T2 (*n* = 2). The fourth malignant lesion was reported to be a T2 laryngeal carcinoma, and the patient underwent TORS with the Flex System for endoscopy and biopsy. The authors reported that lesions of the ventricular fold could not be visualized properly in two patients because of unfavorable narrow anatomy, while the transoral microscopic approach in these two patients was successful. Thus, the Flex System has demonstrated to be a safe and effective device in TORS, but experience in oncologic resection of laryngeal neoplasms is still in the early stages. Furthermore, some technical limitations remain as to adequate endolaryngeal exposure for oncologic surgery, which warrants further technological developments.

## Conclusion

Transoral robotic surgery for laryngeal cancer has been shown to be feasible for minimally invasive partial laryngectomy for either supraglottic or glottic cancer, as well as for total laryngectomy, in selected patients. However, the level of evidence for oncological safety as compared with other conventional treatment modalities remains low due to the small number of published series to date and the lack of randomized trials. Furthermore, it is still to be proved that TORS provide oncological and functional outcomes comparable with TLM. Another major issue with TORS in laryngeal cancer is the underlying necessity for access and adequate exposure, as instrument size and individual patient anatomy can present serious limitations. Future technological developments and miniaturization should improve the feasibility in a larger number of patients.

## Author Contributions

The author confirms being the sole contributor of this work and approved it for publication.

## Conflict of Interest Statement

The author declares no conflict of interest. The article submitted did not involve any personal, professional, or financial relationships that could potentially be construed as a conflict of interest.

## References

[1] Holsinger FC Ferris RL. Transoral endoscopic head and neck surgery and its role within the multidisciplinary treatment paradigm of oropharynx cancer: robotics, lasers, and clinical trials. J Clin Oncol (2015) 33(29):3285–92. 10.1200/JCO.2015.62.3157 26351337 PMC4586168

[2] Weinstein GS O’Malley BW Jr Magnuson JS Carroll WR Olsen KD Daio L Transoral robotic surgery: a multicenter study to assess feasibility, safety, and surgical margins. Laryngoscope (2012) 122(8):1701–7. 10.1002/lary.23294 22752997

[3] Sload R Silver N Jawad BA Gross ND. The role of transoral robotic surgery in the management of HPV negative oropharyngeal squamous cell carcinoma. Curr Oncol Rep (2016) 18(9):53. 10.1007/s11912-016-0541-x 27469262

[4] Weinstein GS Transoral robotic surgery and the standard of care. Int J Radiat Oncol Biol Phys (2017) 97(1):4 10.1016/j.ijrobp.2016.09.028 27979455

[5] Genden EM O’Malley BW Jr Weinstein GS Stucken CL Selber JC Rinaldo A Transoral robotic surgery: role in the management of upper aerodigestive tract tumors. Head Neck (2012) 34(6):886–93. 10.1002/hed.21752 22610591

[6] Smith RV. Transoral robotic surgery for larynx cancer. Otolaryngol Clin North Am (2014) 47(3):379–95. 10.1016/j.otc.2014.03.003 24882796

[7] Succo G Peretti G Piazza C Remacle M Eckel HE Chevalier D Open partial horizontal laryngectomies: a proposal for classification by the working committee on nomenclature of the European Laryngological Society. Eur Arch Otorhinolaryngol (2014) 271(9):2489–96. 10.1007/s00405-014-3024-4 24691854

[8] Rodrigo JP Suarez C Silver CE Rinaldo A Ambrosch P Fagan JJ Transoral laser surgery for supraglottic cancer. Head Neck (2008) 30(5):658–66. 10.1002/hed.20811 18327778

[9] Silver CE Beitler JJ Shaha AR Rinaldo A Ferlito A. Current trends in initial management of laryngeal cancer: the declining use of open surgery. Eur Arch Otorhinolaryngol (2009) 266(9):1333–52. 10.1007/s00405-009-1028-2 19597837

[10] Timon CI Gullane PJ Brown D Van Nostrand AW. Hyoid bone involvement by squamous cell carcinoma: clinical and pathological features. Laryngoscope (1992) 102(5):515–20. 10.1288/00005537-199205000-00008 1573947

[11] Lee NK Goepfert H Wendt CD. Supraglottic laryngectomy for intermediate-stage cancer: U.T. M.D. Anderson Cancer Center experience with combined therapy. Laryngoscope (1990) 100(8):831–6. 10.1288/00005537-199008000-00007 2381259

[12] Laccourreye L Garcia D Menard M Brasnu D Laccourreye O Holsinger FC. Horizontal supraglottic partial laryngectomy for selected squamous carcinoma of the vallecula. Head Neck (2008) 30(6):756–64. 10.1002/hed.20780 18286490

[13] Weinstein GS O’Malley BW Jr Snyder W Hockstein NG. Transoral robotic surgery: supraglottic partial laryngectomy. Ann Otol Rhinol Laryngol (2007) 116(1):19–23. 10.1177/000348940711600104 17305273

[14] Alon EE Kasperbauer JL Olsen KD Moore EJ. Feasibility of transoral robotic-assisted supraglottic laryngectomy. Head Neck (2012) 34(2):225–9. 10.1002/hed.21719 21500308

[15] Olsen SM Moore EJ Koch CA Price DL Kasperbauer JL Olsen KD. Transoral robotic surgery for supraglottic squamous cell carcinoma. Am J Otolaryngol (2012) 33(4):379–84. 10.1016/j.amjoto.2011.10.007 22133967

[16] Lallemant B Chambon G Garrel R Kacha S Rupp D Galy-Bernadoy C Transoral robotic surgery for the treatment of T1-T2 carcinoma of the larynx: preliminary study. Laryngoscope (2013) 123(10):2485–90. 10.1002/lary.23994 23918439

[17] Mendelsohn AH Remacle M Van Der Vorst S Bachy V Lawson G. Outcomes following transoral robotic surgery: supraglottic laryngectomy. Laryngoscope (2013) 123(1):208–14. 10.1002/lary.23621 23008093

[18] Oysu C Sahin-Yilmaz A. En bloc resection of epiglottic tumors with transoral robotic approach—preliminary results. Int J Med Robot (2013) 9(4):477–9. 10.1002/rcs.1516 23728889

[19] Ozer E Alvarez B Kakarala K Durmus K Teknos TN Carrau RL. Clinical outcomes of transoral robotic supraglottic laryngectomy. Head Neck (2013) 35(8):1158–61. 10.1002/hed.23101 22907898

[20] Park YM Kim WS Byeon HK Lee SY Kim SH. Surgical techniques and treatment outcomes of transoral robotic supraglottic partial laryngectomy. Laryngoscope (2013) 123(3):670–7. 10.1002/lary.23767 23288629

[21] Kayhan FT Kaya KH Altintas A Sayin I. Transoral robotic supraglottic partial laryngectomy. J Craniofac Surg (2014) 25(4):1422–6. 10.1097/SCS.0000000000000572 24911606

[22] Perez-Mitchell C Acosta JA Ferrer-Torres LE. Robotic-assisted salvage supraglottic laryngectomy. P R Health Sci J (2014) 33(2):88–90. 24964644

[23] Vicini C Leone CA Montevecchi F Dinelli E Seccia V Dallan I. Successful application of transoral robotic surgery in failures of traditional transoral laser microsurgery: critical considerations. ORL J Otorhinolaryngol Relat Spec (2014) 76(2):98–104. 10.1159/000359953 24801375

[24] Mendelsohn AH Remacle M. Transoral robotic surgery for laryngeal cancer. Curr Opin Otolaryngol Head Neck Surg (2015) 23(2):148–52. 10.1097/MOO.0000000000000144 25692623

[25] Razafindranaly V Lallemant B Aubry K Moriniere S Vergez S Mones ED Clinical outcomes with transoral robotic surgery for supraglottic squamous cell carcinoma: experience of a French evaluation cooperative subgroup of GETTEC. Head Neck (2016) 38(Suppl 1):E1097–101. 10.1002/hed.24163 26435046

[26] Meulemans J Vanclooster C Vauterin T D’Heygere E Nuyts S Clement PM Up-front and salvage transoral robotic surgery for head and neck cancer: a Belgian multicenter retrospective case series. Front Oncol (2017) 7:15. 10.3389/fonc.2017.00015 28232904 PMC5298968

[27] Durmus K Gokozan HN Ozer E. Transoral robotic supraglottic laryngectomy: surgical considerations. Head Neck (2015) 37(1):125–6. 10.1002/hed.23645 24616067

[28] Muderris T Bercin S Sevil E Acar B Kiris M. Transoral robotic surgery for atypical carcinoid tumor of the larynx. J Craniofac Surg (2013) 24(6):1996–9. 10.1097/SCS.0b013e3182a28c2c 24220389

[29] Solares CA Strome M. Transoral robot-assisted CO2 laser supraglottic laryngectomy: experimental and clinical data. Laryngoscope (2007) 117(5):817–20. 10.1097/MLG.0b013e31803330b7 17473675

[30] Cernea CR Matos LL de Carlucci Junior D Leonhardt FD Haddad L Walder F Transoral robotic supraglottic partial laryngectomy: report of the first Brazilian case. Braz J Otorhinolaryngol (2016). 10.1016/j.bjorl.2016.01.016 PMC9452258 27269129

[31] Ansarin M Zorzi S Massaro MA Tagliabue M Proh M Giugliano G Transoral robotic surgery vs transoral laser microsurgery for resection of supraglottic cancer: a pilot surgery. Int J Med Robot (2014) 10(1):107–12. 10.1002/rcs.1546 24288345

[32] De Virgilio A Park YM Kim WS Baek SJ Kim SH. How to optimize laryngeal and hypopharyngeal exposure in transoral robotic surgery. Auris Nasus Larynx (2013) 40(3):312–9. 10.1016/j.anl.2012.07.017 23083625

[33] Park YM Byeon HK Chung HP Choi EC Kim SH Comparison of treatment outcomes after transoral robotic surgery and supraglottic partial laryngectomy: our experience with seventeen and seventeen patients respectively. Clin Otolaryngol (2013) 38(3):270–4. 10.1111/coa.12101 23441587

[34] Dziegielewski PT Kang SY Ozer E. Transoral robotic surgery (TORS) for laryngeal and hypopharyngeal cancers. J Surg Oncol (2015) 112(7):702–6. 10.1002/jso.24002 26266762

[35] Lawson G Mendelsohn AH Van Der Vorst S Bachy V Remacle M Transoral robotic surgery total laryngectomy. Laryngoscope (2013) 123(1):193–6. 10.1002/lary.23287 22522233

[36] Dowthwaite S Nichols AC Yoo J Smith RV Dhaliwal S Basmaji J Transoral robotic total laryngectomy: report of 3 cases. Head Neck (2013) 35(11):E338–42. 10.1002/hed.23226 23471833

[37] Smith RV Schiff BA Sarta C Hans S Brasnu D. Transoral robotic total laryngectomy. Laryngoscope (2013) 123(3):678–82. 10.1002/lary.23842 23299907

[38] Krishnan G Krishnan S. Transoral robotic surgery total laryngectomy: evaluation of functional and survival outcomes in a retrospective case series at a single institution. ORL J Otorhinolaryngol Relat Spec (2017) 79(4):191–201. 10.1159/000464138 28609773

[39] Gorphe P Von Tan J El Bedoui S Hartl DM Auperin A Qassemyar Q Early assessment of feasibility and technical specificities of transoral robotic surgery using the da Vinci Xi. J Robot Surg (2017) 11(4):455–61. 10.1007/s11701-017-0679-z 28064382

[40] Sjogren EV. Transoral laser microsurgery in early glottic lesions. Curr Otorhinolaryngol Rep (2017) 5(1):56–68. 10.1007/s40136-017-0148-2 28367361 PMC5357474

[41] Hartl DM Ferlito A Brasnu DF Langendijk JA Rinaldo A Silver CE Evidence-based review of treatment options for patients with glottic cancer. Head Neck (2011) 33(11):1638–48. 10.1002/hed.21528 21990228

[42] Peretti G Piazza C Mora F Garofolo S Guastini L. Reasonable limits for transoral laser microsurgery in laryngeal cancer. Curr Opin Otolaryngol Head Neck Surg (2016) 24(2):135–9. 10.1097/MOO.0000000000000240 26963672

[43] O’Malley BW Jr Weinstein GS Hockstein NG. Transoral robotic surgery (TORS): glottic microsurgery in a canine model. J Voice (2006) 20(2):263–8. 10.1016/j.jvoice.2005.10.004 16472973

[44] Park YM Lee WJ Lee JG Lee WS Choi EC Chung SM Transoral robotic surgery (TORS) in laryngeal and hypopharyngeal cancer. J Laparoendosc Adv Surg Tech A (2009) 19(3):361–8. 10.1089/lap.2008.0320 19405798

[45] Blanco RG Ha PK Califano JA Saunders JM. Transoral robotic surgery of the vocal cord. J Laparoendosc Adv Surg Tech A (2011) 21(2):157–9. 10.1089/lap.2010.0350 21323600

[46] Vural E Tulunay-Ugur OE Suen JY. Transoral robotic supracricoid partial laryngectomy with cartilaginous framework preservation. J Robot Surg (2012) 6(4):363–6. 10.1007/s11701-012-0349-0 27628480

[47] Kayhan FT Kaya KH Sayin I. Transoral robotic cordectomy for early glottic carcinoma. Ann Otol Rhinol Laryngol (2012) 121(8):497–502. 10.1177/000348941212100801 22953654

[48] Wang CC Liu SA Wu SH Lin WJ Jiang RS Wang L. Transoral robotic surgery for early glottic carcinoma involving anterior commissure: preliminary reports. Head Neck (2016) 38(6):913–8. 10.1002/hed.24354 26714200

[49] Remacle M Prasad VMN. Preliminary experience in transoral laryngeal surgery with a flexible robotic system for benign lesions of the vocal folds. Eur Arch Otorhinolaryngol (2018) 275(3):761–5. 10.1007/s00405-018-4900-0 29417276

[50] Hans S Badoual C Gorphe P Brasnu D. Transoral robotic surgery for head and neck carcinomas. Eur Arch Otorhinolaryngol (2012) 269(8):1979–84. 10.1007/s00405-011-1865-7 22143583

[51] Mandapathil M Greene B Wilhelm T. Transoral surgery using a novel single-port flexible endoscope system. Eur Arch Otorhinolaryngol (2015) 272(9):2451–6. 10.1007/s00405-014-3177-1 25018060

[52] Mandapathil M Duvvuri U Guldner C Teymoortash A Lawson G Werner JA Transoral surgery for oropharyngeal tumors using the Medrobotics((R)) Flex((R)) System—a case report. Int J Surg Case Rep (2015) 10:173–5. 10.1016/j.ijscr.2015.03.030 25853845 PMC4430123

[53] Remacle M Prasad V Lawson G Plisson L Bachy V Van der Vorst S Transoral robotic surgery (TORS) with the medrobotics flex system: first surgical application on humans. Eur Arch Otorhinolaryngol (2015) 272(6):1451–5. 10.1007/s00405-015-3532-x 25663191

[54] Mattheis S Hasskamp P Holtmann L Schafer C Geisthoff U Dominas N Flex robotic system in transoral robotic surgery: the first 40 patients. Head Neck (2017) 39(3):471–5. 10.1002/hed.24611 27792258

[55] Lang S Mattheis S Hasskamp P Lawson G Guldner C Mandapathil M A European multicenter study evaluating the flex robotic system in transoral robotic surgery. Laryngoscope (2017) 127(2):391–5. 10.1002/lary.26358 27783427

